# Mexican American Intergenerational Research: Transformative Model of Occupational Therapy

**DOI:** 10.1155/2024/6301510

**Published:** 2024-07-09

**Authors:** Maritza Montiel Tafur, Yvonne de la Torre Montiel, Miguel Montiel

**Affiliations:** ^1^ The University of Utah, Salt Lake, USA; ^2^ South Mountain Community College, Phoenix, USA; ^3^ Arizona State University, Tempe, USA

## Abstract

Thirty-seven interviews of Mexican American women who crossed the border into the United States during the era of the Mexican Revolution of 1910 were analyzed using constructivist grounded theory methods. The intent is to expand the occupational therapy profession's occupational consciousness and cultivate cultural humility. Four themes emerged from the data: suffering, work, yearning for an education, and compassion for others. The findings suggest that environmental barriers such as hierarchy (patriarchy and discrimination) and physical barriers (limited access to built environments, lack of nonexploitative work opportunities, and hostile educational institutions) prevented occupational participation. Small acts of resistance through everyday living (finding joy, playing, self-sufficiency, and community organizing) were identified as facilitators of occupational participation. The research findings challenge proposed assumptions found within the occupational therapy literature: (1) humans and occupations exist as separate from their environments, and (2) work, productivity, and leisure contribute positively to health. The Transformative Model of Occupational Therapy is introduced as a decolonized framework that inextricably links individual health to community and global health. The model centers play, social participation, work, and education as occupations that contribute to the common good. These occupations are kept in equilibrium within the Four Pillars of Culture (self-determination, compassion, sustainability, and language) or the cultural values identified and derived from the stories.

## 1. Introduction

### 1.1. Positioning

Maritza Montiel Tafur (first author): I am a fifth-generation Arizona native and live in the ancestral homelands of the Akimel O'odham, Onk Akimel O'odham, Tohono O'odham, and the Ak-Chin peoples. My maternal ancestors lived in the Mexican territory now known as Arizona before it was acquired by the United States in 1848. My paternal ancestors fled the Mexican Revolution and came to work in the United States throughout the early part of the 20th century. I identify as Mexican American and Xican@ and recognize my ancestors as both European and Indigenous peoples, but I am not a registered tribal member of any group. I am a board-certified pediatric occupational therapist and work in the public education setting. I offer this work in honor of my ancestors and as part of a greater movement to broaden and enrich occupational therapy.

Miguel Montiel and Yvonne de la Torre Montiel (second and third authors) are my parents and professors emeritus from Arizona State University (ASU) and South Mountain Community College in Arizona, United States, respectively. They were responsible for the data collection for this research, later published in the book, *World of Our Mothers: Mexican Revolution–Era Immigrants and Their Stories* [1]. Miguel Montiel received his doctorate from the University of California (UC), Berkeley. Yvonne received her teaching credentials from UC Berkeley and later earned a PhD from ASU in Elementary Education. They live in Phoenix, Arizona, where they continue their activism and research.

### 1.2. Decolonizing Occupational Therapy

Colonization began in the 15th century and refers to the European political domination and violent dispossession of land throughout the Americas, Australia, Africa, and parts of Asia [2]. The papal decree, Doctrine of Discovery (1493), justified the European appropriation of Indigenous ancestral lands, claiming that Christians were culturally and morally superior and therefore entitled to dominate and subjugate non-Christian lands and peoples [3]. The exploitation of people and land ensued. Forced labor aided the colonizers in gaining wealth and privilege in the so-called New World. An estimated 55 million Indigenous people died from violence and disease, nearly 90% of the population, in the period after Europeans invaded the Americas in 1942 [4]. Despite this historical trauma, contemporary Indigenous peoples of the Americas have endured by preserving, protecting, and reclaiming their languages and traditions [5].

Groups of people who suffered from the negative impacts of colonialism continue to have limited access to resources, while those who often identify with the colonizers have an overabundance. For example, the richest 1% of the world's population is responsible for more than twice the global emissions than that of the poorest 50% [6]. This inequity is a barrier to occupational participation and contributes to the disproportionate health burdens that exist among minority groups who experience greater rates of death, disability, and disease compared with non-Hispanic White people [7, 8]. An example of this health disparity can be found in Native American peoples, whose rates of type II diabetes mellitus (T2DM) are 3.2 times higher than all other ethnic groups in the United States combined [9]. Diabetes was rare among Native Americans before 1940; however, groups who experienced displacement, loss of subsistence economies, and starvation during the “postcolonial” era are now more susceptible to developing T2DM than Native American groups who did not suffer from this pattern of historical trauma [10]. This puts into question theories that indicate certain “racial” groups are somehow genetically predisposed to inheriting diabetes and forces practitioners to consider the impact of environmental factors such as violence and colonization on health [11, 12].

According to Hammell [13], the historical influence of colonialism on occupational participation is often ignored. Hammell [13] calls for practitioners to critically self-reflect on the following colonial ideologies often perpetuated within occupational therapy theory: (1) Individuals exist as separate from their environments. (2) Individuals seek mastery over the environment. (3) Independence is an ideal, while dependence/interdependence is a pathology [14–17]. Visual models used within occupational therapy have also been criticized for promoting individualism, independence, and mastery over the environment [18, 19]. For example, the Person-Environment-Occupation-Performance (PEOP) model is represented by a Venn diagram (a series of overlapping circles and ovals) and proposes that occupational performance can be enhanced by maximizing the balance between the person and environment. Although there is some overlap, visually, this model indicates the person as distinct from their environment by placing the person (oval) in opposition to or contrary to the environment (oval). This suggests adversity and promotes a binary dualism of thought (person versus the environment). Dependence on the environment and interconnection with others is not implied through this representation. Reid et al. [19] further indicate that this visual representation promotes the unsustainable ideology that people's occupation is created when humans seek mastery over their environment.

Ramugondo [20] introduces the language of occupational consciousness as a self-reflective approach to resisting hegemony in healthcare, or the dominance of biomedical models and the corporatization of medicine [21]. Occupational consciousness can promote a practitioner's awareness of how everyday living may reinforce or dismantle established colonial ideologies. Occupational consciousness requires cultural humility to avoid cultural imperialism, a mode that regards the dominant group as the standard by which others are judged [22]. Cultural humility involves a critical self-reflection of our assumptions and how our own positioning impacts therapeutic relationships and power dynamics. Through this consciousness-raising process, practitioners can reclaim the legitimacy of knowledge found within historically marginalized groups.

### 1.3. Literature Review

The American Occupational Therapy Association's (AOTA) 2025 vision is to be “an inclusive profession” that “maximizes health, well-being, and quality of life for all people, populations, and communities through effective solutions that facilitate participation in everyday living” [23]. To demonstrate AOTA's diversity, equity, and inclusion efforts, photographs of Black, Indigenous, and people of color (BIPOC) are represented on the website. The occupational therapy profession within the United States, however, does not reflect this diversity and remains a predominantly White profession (82.5%) [24]. For example, although Hispanics comprise 19% of the total U.S. population, they represent 4%-8% of practitioners and students [25, 26]. The same trend can be seen across all ethnic groups except for Asians, who represent 5%-7% of practitioners/students and 6% of the total U.S. population [25, 27].

The lack of diversity within the profession limits the potential to support all people in pursuit of health-sustaining occupations [23]. Fostering diverse academic environments leads to greater public health for underserved populations. Clients who share a similar cultural background as their providers achieve greater health outcomes [28, 29].

### 1.4. OT Literature on Mexican Americans

There is limited research on Mexican American populations living in the United States. Tafur et al. [30] outline traditional healing remedies and provide a historical background on the practice of *curanderismo.* Folk-based remedies are introduced as a culturally relevant self-care occupation that therapists can support to promote a greater quality of life. Much of the more recent research on Mexican American populations is focused on decreasing “nonadherent” behavior in Mexican Americans with type II diabetes mellitus (T2DM) or end-stage renal disease resulting from unmanaged T2DM [31–34]. Participants in these studies are Mexican Americans living near the Texas-Mexico border, where there are particularly high incidences of T2DM. The focus of the research is on understanding the cultural perspectives of T2DM to design culturally specific interventions. Findings suggest facilitating an internal locus of control, increasing peer interactions, and encouraging family support as ways to create culturally specific interventions for this population [31, 33, 34]. None of the articles mentions the historical or environmental factors that may contribute to the high incidences of diabetes among the population living in this geographical area.

Deficit-based language that focuses on “nonadherent” and “noncompliant” behaviors may inadvertently perpetuate stereotypes of Mexican Americans being passive or lazy. Culturally specific concepts to describe Mexican families are mentioned including *machismo*, *fatalismo*, and *familismo. Machismo* refers to patriarchal family dynamics that emulate the hierarchical structures found mainly within the Catholic Church. *Machismo* assumes the authority of the male members of the household, typically the father, who is believed to be the main provider and protector of the family. *Fatalismo* is the fatalistic belief that God is ultimately in control of a person's destiny and implies an external locus of control. *Familismo* places the family at the center of importance and is a motivator to be a productive, accountable member of the family and society [34]. These concepts lack the nuances of Mexican American culture and serve to promote stereotypes of the domineering, crude patriarch; the martyred, docile mother; and the obedient, fearful child. Currently, there is a lack of literature that incorporates a strength-based approach on how Mexican Americans' unique perspectives and experiences can inform occupational therapy theories and practices.

### 1.5. Historical Background of Mexican Americans

The Mexican-American War was fought from 1846 to 1848 after a series of disputes along the Rio Grande resulted in the signing of the Treaty of Guadalupe Hidalgo (1848), whereby California, Utah, Arizona, New Mexico, Colorado, Texas, Oklahoma, and Nevada became U.S. territories [35]. The forceful acquisition of these lands was influenced by the Doctrine of Discovery and rationalized by Manifest Destiny (1812-1867), or the belief that the United States was divinely ordained to expand westward to settle the entire North American continent [3, 36]. The systematic removal of Indigenous peoples from their lands, separating children from their parents and sending them to government schools in attempts to assimilate and “Americanize” them, was considered justifiable, along with imposing Christianity to ensure their salvation. Through the colonial settler lens, Native Americans, who practiced land stewardship, were wasting an opportunity to generate wealth by leaving the land undeveloped. During this period of western expansion, water resources were diverted from Indigenous communities, and agricultural and hunting practices were disrupted, leading to food scarcity and starvation for many Native Americans. Native peoples resisted in a series of revolts throughout the 17th and early 19th centuries.

Slavery had extended west to Texas, and disagreements about expanding slavery to the newly acquired southwest eventually contributed to the Civil War in 1861. The Emancipation Proclamation (1863) did not apply to Native Americans who were not considered citizens until 1924 [37]. Many native people were enslaved, forced into debt peonage, or leased as convicts (a system where prisoners were leased as workers for private companies for no pay). The building of the transcontinental railroads in the late 1800s brought a swell of White American settlers to the Southwest. During this time, many Mexicans living in the Southwest lost their lands or were displaced. By the late 1800s, White Americans came to own four-fifths of the once Mexican-owned lands. Mexicans who lost their lands were relegated to working dangerous and low-paying jobs that served the economic interests of the settlers, predominately in agriculture and mining [38]. The Mexican Revolution of 1910 created an opportunity to exploit Mexican workers fleeing from the violence of the revolution, where an estimated one million Mexican people were killed. During this early period of 20th-century migration into the Southwest, border crossings were relatively fluid. It was not until 1924, with the establishment of the U.S. Border Control Agency, that Mexican immigration was regulated.

## 2. Methods

The research question is as follows: How can the stories of Mexican American women born in the late 19th and early 20th centuries expand our occupational consciousness and cultivate the occupational therapy profession's cultural humility?

Qualitative, grounded theory methods were selected for this research because the intention was to provide insight into a topic where little research has previously been done [39]. A grounded theory approach is complementary to decolonized research methodologies outlined by Smith [40] and applies the following: (1) critical reflexivity, a process by which the researcher examines how their own positioning impacts the interpretation and response to the data; (2) respect for the participants in the study and establishing a reciprocal relationship; (3) honoring Mexican American identity and ways of knowing; and (4) generating a theory of occupational therapy based on the women's stories [40, 41].

### 2.1. Data Generation

The raw data for this study involved interviews conducted with 37 Mexican and Mexican American women who were born between 1881 and 1927. Three women were born in the United States (Arizona, Colorado, and Texas). Thirty-four women were born throughout Mexico (Sonora, Aguascalientes, Guanajuato, Jalisco, Chihuahua, Sinaloa, Coahuila, Durango, Zacatecas, Mexico City, Nuevo Leon, Tlaxcala, Tamaulipas, and Baja California). They crossed legally and illegally into the United States between 1908 and 1965, at different points of entry, (El Paso, Laredo, Nogales, Douglas, Calexico, and Andrade). Some were sojourners and journeyed back and forth between countries, but eventually settled in the Southwest.

The interviews were conducted between 1974 and 1975 by Miguel Montiel, Yvonne de la Torre Montiel, and their colleagues. The interviews were originally intended to promote understanding. The women were selected and interviewed based on a convenience sample and their willingness to share their stories. The interviews were recorded in Spanish, transcribed, translated into English, and edited into readable form. The interviews followed a general format of answering open-ended questions: What was your life like in Mexico? How did you cross the border? And what was your life like in the United States? The stories were published almost 50 years later in the book, *World of Our Mothers: Mexican Revolution–Era Immigrants and Their Stories* [1].

### 2.2. Data Analysis

Thirty-seven interviews were analyzed using grounded theory methods, first developed by Glaser and Strauss [42], and influenced by a constructivist grounded theory approach developed by Charmaz [39]. The initial steps involved a constant comparative analysis and an open coding process in which simple words or codes were attributed to the prominent features of the women's stories. The continuous comparison allowed for patterns to emerge from the data, and smaller codes were collapsed into broader categories. To understand the data from an occupational therapy perspective, the categories were organized using the Person-Environment-Occupation (PEO) model, a client-centered model used within occupational therapy practice that recognizes the transactional relationships between the person, environment, and occupation [43]. The greater the congruence between the person, environment, and occupation, the greater the propensity towards participation in desired occupations [43]. In practice, the PEO model can be used to determine facilitators and barriers to occupational participation. The data was then organized chronologically into categories of childhood, adolescence, adulthood, and elderhood to give a lifespan perspective. The final step involved selective coding, where themes were identified, compared, and analyzed based on their relevance to the research question. The first author kept a record of their thoughts, feelings, and reactions through memos and journaling and traced the reasons why categories and themes were selected. The findings were then used to generate theory.

#### 2.2.1. Resolana

*Resolana* is a Chicanx/Mexican American/Indo-Hispano cultural practice in which *resolaneros*, or members of the community, gather and talk. According to Tomás Atencio (founder of *La Academia de la Nueva Raza*), *resolana* has a dual meaning and is both a culturally specific practice in which knowledge is generated through dialogue and a physical place where the sun reflects on exterior walls and generates warmth [44]. Within a *resolana*, people can raise their critical consciousness [45] by sharing ideas in a safe space while authenticating the cultural knowledge derived from the connection (and sometimes dissonance) with others. The knowledge derived through this process is referred to as *el oro del barrio*. *Oro* is the gold that is precious, shines, and reflects the sun. The *barrio* is the community and neighborhood where people live and work. Regular *resolanas* occurred throughout the research process to identify themes, triangulate the data, gain deeper insight into the women's stories, and reflect upon our relationship with them [44]. *Resolanas* are proposed as a culturally relevant, decolonizing methodology in which people generate wisdom and recognize their agency through connection with others.

## 3. Findings

### 3.1. Themes

Four themes emerged from the stories: suffering, work, yearning for an education, and compassion for others.

#### 3.1.1. Suffering

Women witnessed the violence of the Mexican Revolution, and many saw dead bodies stacked upon one another in the streets. Soldiers looted, pillaged, and raped. Families fled the violence and traveled north to the United States by horse/mule-drawn wagons or trains. Some experienced hunger along their journey. Despite their trauma, a woman recalled joy and play during her long journey into the United States because her family was together. Families were recruited to work in agriculture and recalled being treated like livestock by labor recruiters and bosses. Families who worked in agriculture lived in encampments and shacks, with limited access to clean drinking water or blankets to keep them warm. People were not permitted to leave the camps for supplies. Families struggled to make a living because of poor pay and intermittent seasonal work.

The women recalled child abuse. At 6 years old, Inés worked as a slave in her grandparents' home and would get beaten if she did not meet their cleaning expectations. Refugia's father would beat her if she talked to boys. She buried notes and gifts from boyfriends to avoid the beatings. Children were spanked and shamed in schools by teachers. Husbands and fathers were controlling, and friendships and socializing were forbidden.

Women experienced discrimination. Neighborhoods, theaters, dance halls, and public pools were racially segregated. When Mexican Americans served in the U.S. military and returned from World War II, things changed, and people organized and protested the unfair treatment. Women were called greasers or “Mexican pigs” and were treated with disgust and hatred by White Americans. Lighter skin made it easier to pass. Mexicans rarely secured leadership positions and were frustrated that better jobs and pay went to White Americans. Women were denied pensions or laid off before they were eligible to receive them. Women were abused and exploited at work and frequently moved from job to job in search of better opportunities.

Women experienced terrible sorrow. Husbands and fathers died in mining accidents, drunken brawls, or abandoned their families. One woman lost five of her children to pneumonia and explained that she was poor and did her best to keep them warm by stoking the fire.

#### 3.1.2. Work

When men abandoned their families, women became the primary providers, responsible for earning money, child-rearing, financial management, home management, meal preparation, cleanup, and shopping. Mothers and daughters worked together to sustain their households. Older daughters cared for younger siblings. Mothers taught their daughters to be self-sufficient and taught them many skills to make ends meet (cooking, domestic work, sewing, and farmwork). As a child, Fina had many chores, but what she wanted most was to play with the neighbor's dog. She snuck play so her mother did not catch her because her mother thought play was nonsense. Children did not have an opportunity to play or make friends because they were always working.

Women showed self-determination and expressed self-confidence. Enriqueta described herself as “decisive and not afraid” (Montiel & de la Torre Montiel, p. 221). She explained, “I never say no, even if I don't know. I feigned competence and said I was experienced at everything” (Montiel & de la Torre Montiel, p. 223). Women worked multiple jobs, from “sunrise to sunset” (Montiel & de la Torre Montiel, p. 121). Many women described themselves as working like men. Most of the women cared for large families, one woman had 22 children. The work of caring for a large family with limited resources was significant. Because women worked so tirelessly, they enjoyed few leisure activities. People attended dances even when they had little food and their shoes were falling apart.

#### 3.1.3. Yearning for Education

The women held strong beliefs in education; one woman described education as “everyone's salvation” (Montiel & de la Torre Montiel, p. 34). Access to schools in Mexico was limited, especially for children who lived in rural villages. Some women never attended school because there was little to no transportation, and some families felt their girls should get married instead. Eight-year-old Fina pleaded with her mother to go to school. When her mother finally relented, she insisted Fina do her chores before school, which often made her late. She recalled being spanked by teachers for being late, and she described these events as demoralizing. Fina eventually dropped out. Inés was sent to kindergarten as an adolescent girl going through puberty. She went to school with much younger children who ridiculed her and eventually refused to go. Women lamented never going to school because the family was always working. Carolina explained, “I never got a chance to even pick up a pencil or have someone teach me anything. It was difficult. It saddens me to think of how I spent my life…” (Montiel & de la Torre Montiel, p. 264). The women often called themselves *burras* (stupid) because they did not have a formal education or did not speak English. Children were punished for speaking Spanish at school (wearing tape over their mouths, being forced to wear a dunce cap, and being slapped). Mexicans did not want to challenge the teachers because they could be thrown in jail.

#### 3.1.4. Compassion for Others

Women prioritized the well-being of others over riches or luxuries. Women used the extra money they had to buy food and distribute it to the hungry. Many women described how Mexican people shared food with each other and were quick to help those in need. They had little time to be involved in church but raised money by preparing food to sell. Women believed having inner moral values was more precious than money. The women expressed gratitude to those who had helped them, including their White allies. A woman reflected on her life as an older adult, “I live like a queen. I am supported. I am free to move about. I close my doors very early and watch television. What else could I want? I pay the rent for this little house. I am grateful to *Gringos*. I am now happy with my life after suffering so much” (Montiel, de la Torre Montiel, p. 108). Because many Mexican Americans did not have access to common social benefits, they formed nonprofit mutual aid societies where members paid dues and were given benefits for medical care, funerals, and unemployment. After entering the United States, many women sought refuge with family or friends but expressed fear of being a burden. These feelings extended to a reluctance to accept welfare.

Women believed in service to their country and were proud of their children who served in the U.S. military. The women saved and invested money, bought homes, started businesses, built churches, organized their communities for access to resources (sanitation and water), got their citizenship, got involved in politics, and voted.

### 3.2. Discussion

Occupational consciousness requires an awareness of the systems that sustain and dismantle unequal access to desired occupations [20]. Of the environmental factors that sustain inequality, none are more dominant and pervasive than hierarchical social systems. Within a social hierarchy, the “other” is ranked according to their perceived status or authority. Power is abused. People are not understood as equal, they are exploited, their contributions are marginalized, they are made invisible, and they are deemed less worthy. Racial hierarchies placed people of color as inferior. The women remembered being treated poorly because of their immigration status or having dark skin. Mexicans were dehumanized and regarded as animals, and access to public spaces (theaters and public pools) was restricted. Landowners took advantage of desperate families for their own financial gain. Children were forced into manual labor to help contribute to the economic family unit. The work resembled peonage, where workers had minimal rights, lived in encampments (shacks), did not have access to clean water, experienced food insecurity, and had limited opportunities to advance financially. Gender hierarchies or patriarchy authorized men to leave their families, placing the financial and emotional burden on their wives and children. Fathers felt entitled to physically abuse their children and restrict their friendships.

Despite yearning for an education, environmental barriers such as limited access to nearby schools prevented children from attaining an education. Social barriers also forced children to drop out of school because of hostile educational institutions where children were shamed and experienced corporal punishment for speaking Spanish, and Mexican families were treated with disgust. Lack of sustainable work opportunities was a barrier to pursuing both play and education because low-paying jobs forced children to work and help support their families rather than play and go to school.

Cultural humility involves critical self-reflection to enhance our capacity to engage in a reflexive process and rectify the ways therapists have reinforced unjust power dynamics [46]. Occupational therapists maintain unjust power dynamics by promoting independence and encouraging people to “overcome” their “physical and mental impairments” ([47], p. 622). This deficit discourse is problematic because it creates a hierarchy where someone who overcomes and achieves independence is considered more valuable than someone who is dependent upon others, struggles financially, or exists with a disability or illness. The stories show that education and play participation were hindered because of a lack of environmental and social support and not because of the women's lack of independence, shortcomings, or failures. The women worked hard and were self-reliant but lacked the social support to achieve the occupational participation they desired.

Raising occupational consciousness requires a critical awareness of the facilitators of occupational participation and the ways in which people dismantle systems of oppression through their everyday occupations. Resistance to hegemony through everyday living or small acts of resistance was demonstrated throughout the women's stories [48]. Mexican communities prioritized social participation and formed mutual aid societies where they collected and shared funds for the purposes of connecting people with needed resources for funeral expenses, housing, food, or healthcare. Despite poverty and not having shoes or nice clothes, people found joy in going to dances and socializing. Children sneak play in their backyards or accept gifts from boys (and bury them) despite their parent's disapproval and probable beatings. Regardless of experiencing hardships and trauma, children played and had fun on the long journey north with their parents. Women find happiness, described as living in peace, in a modest, clean home. Despite discrimination, women found ways to educate their children, get their citizenship, vote, organize their communities, save and invest money, buy homes, start businesses, and get involved in politics. Women defied the gender norms of their time by acting as heads of households. They were dedicated to helping others, gave food and money to those in need, shared resources, and showed gratitude despite their suffering. In a society that discouraged people from speaking their native language, preserving the Spanish language and learning English were acts of resistance. Occupational therapists can identify these small acts of resistance and support their clients in exercising their agency and self-determination through everyday living.

The stories demonstrate the need to practice cultural humility and to reconsider the assumption that participation in self-care, productivity, and leisure contribute positively to health [46]. The drudgery involved in many of the productive, paid, and unpaid occupations gave little time for leisure and self-care. Occupational therapists must be critical of the historical underpinnings associated with the prioritization of leisurely participation. For example, slavery gave the southern, upper class the prerogative to deny the responsibility of manual labor, which allowed discretionary time to devote to leisure (and education). To them, slavery and exploitation, or delegating labor responsibilities to the lower classes, were seen as movements towards progress [49]. Instead of leisure and self-care, the women prioritized play, social participation, work, and education.

The literature on happiness aligns with parts of the Occupational *Therapy Practice Framework: Domain and Process*, 4th Edition [25, 50] and can guide therapists to focus on research-based occupations that promote health, happiness, and longevity of life. Activities categorized under instrumental activities of daily living (IADL), such as child-rearing, health management/maintenance (nutritious diets/physical activity), religious/spiritual expression, and ensuring our clients have proper rest and sleep, have been shown to promote happiness and well-being ([25, 50]; Harvard Health [51–56]). Occupations that the women prioritized, such as play and social participation, should also be given greater emphasis, as research has shown that these activities are positive contributors to happiness, health, and well-being [25, 50, 57, 58]. Research by Lyunbomirsky et al. [59] indicates that having meaningful relationships, practicing gratefulness, helping others, being optimistic about the future, savoring pleasure, making physical activity a habit, practicing spirituality/religion, and being committed to life goals are what contribute positively to health and happiness. The women's engagement in these activities may explain their seeming contentment with life despite their suffering and difficult circumstances.

### 3.3. Theory

Theory was conceptualized using two-eyed seeing, an Indigenous framework first introduced to occupational therapy literature by Fijal and Beagan [18]. Two-eyed seeing was developed by Mi'Kmaw (Canadian, First Nations) elder Albert Marshall to facilitate a complementary relationship between allopathic medicine and Indigenous knowledge [18]. Two-eyed seeing is a way to serve the common good by developing a mutual understanding and respect between two valuable and distinct worldviews and by learning to see from two perspectives (two eyes) simultaneously. This nonbinary approach can give us sight beyond our eyes and into our hearts, allowing us to see multiple perspectives simultaneously. Within this cosmovision, there are many ways to describe the same phenomenon; each facet of truth is both distinct and interconnected. The first author applied two-eyed seeing by weaving Indigenous/Xicanx perspectives together with the women's cultural values and integrating this knowledge within a complimentary occupational therapy framework.

#### 3.3.1. The Transformational Model of Occupational Therapy

**Figure 1:**
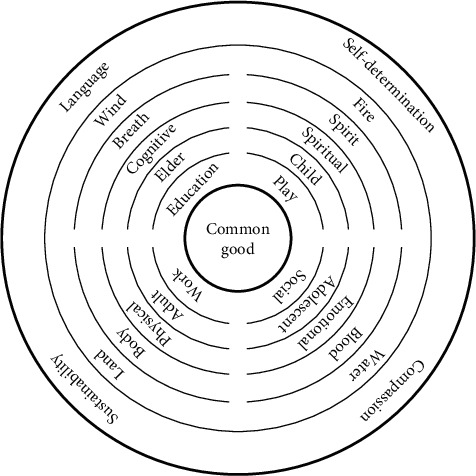
The Transformative Model of Occupational Therapy (TMOT). Note: Image is created and designed by Maritza Montiel Tafur.

Figure 1 shows the Transformative Model of Occupational Therapy (TMOT), the visual representation of the theory derived from this research and inspired by the seven-generation concentric circular genogram proposed by Michiel-Derksen [60]. The number seven evokes the seven future and past generations that influence us today and that we must consider in creating and maintaining a sustainable future [61]. This visual representation also reflects the intergenerational nature of this research and the more than 3 generations that were involved in generating, collecting, and analyzing the data. The circle was chosen because it inspires heterarchical (nonhierarchical) relationships, as each point along the radius is equidistant from the center [60]. The center circle is labeled the common good. The common good also represents the sun; it elicits warmth, illuminates our path, and reminds us that we are part of a larger universe [62]. This circular model indicates that relationships between communities, occupations, and environments are transactional and mutually influential [14, 18]. There is no representation of the individual in this model, but there is recognition that living beings are interdependent and inextricably linked to each other and to nature, an ecological perspective Salmón [63] refers to as kincentric.

The circle is divided into four directions, as seen in the medicine wheel used by many Indigenous peoples to organize philosophical and spiritual concepts [64]. The four groupings are as follows: health-sustaining occupations (play, social participation, work, and education), stages of life (childhood, adolescence, adulthood, and elderhood), dimensions of health (spiritual, emotional, physical, and cognitive), human qualities (spirit, blood, body, and breath), the elements (fire, water, land, and wind), and the Four Pillars of Culture, or the cultural values derived from the women's stories. (self-determination, compassion, sustainability, and language).

The four occupations were derived from the stories and chosen because they were given the most priority in women's lives. Leisure, self-care, and productivity were intentionally omitted because the data indicates these occupations do not contribute positively to health. The stages of life (childhood, adolescence, adulthood, and elderhood) are frequently represented in the medicine wheel and are compatible with the lifespan perspective within occupational therapy. The dimensions of health (spiritual, emotional, physical, and cognitive) are frequently represented in the medicine wheel and are areas that occupational therapists assess to determine client factors, performance factors, and performance skills that support or hinder occupational performance and participation [25, 50]. The human qualities (spirit, blood, body, and breath) are commonly found in the medicine wheel and are life-sustaining functions shared by living beings. The four elements (fire, water, land, and wind) are frequently represented in the medicine wheel and are the building blocks needed to sustain life on Earth. The Four Pillars of Culture (self-determination, compassion, sustainability, and language) are derived from this research.

The Four Pillars of Culture was a term imagined by the first author, derived from research, and represents the cultural values the women shared. The Four Pillars of Culture correspond to each of the four quadrants of the TMOT. The imagery of a pillar reflects the woman's stability or cultural foundation. Like a pyramid, the more stability at the foundation, the more possibility there is for mobility, transcendence, and transformation. When each of the Four Pillars of Culture is practiced and integrated throughout our lifespan, they can facilitate transformative occupational experiences that serve the common good for the next seven future generations. The model defines human well-being as connected to ecological well-being [65]. Cultivating a collective culture in harmony and alignment with individual, community, and global health is intended by this theory. The model should be imagined as dynamic, spiraling, and transactional. These values are not culturally specific but cross-cultural and can be shared by all to cultivate unity and stability.

Each of the Four Pillars of Culture and their corresponding quadrants will be described in the following section. It will begin with a brief description of how each pillar relates to the women's stories and how this impacts global and individual health today. Occupational therapy perspectives and Indigenous wisdom and philosophy were used to inform these concepts.

*(1) Pillar 1: Self-Determination (Play, Childhood, Spiritual, Spirit, and Fire)*. Women succeed and find joy despite their difficult circumstances. Children desired play, but their lives were dominated by work. Play was restricted by their parents and was not valued. Children showed self-determination by sneaking play. People went to dances despite being poor.

Self-determination refers to a person's fundamental right to determine the course of their own lives and to define “ourselves to ourselves” ([44], p. 8). Play refers to intrinsically motivating activities that are freely chosen (self-determined), open-ended, and can have the potential to suspend reality. This can include celebrating, risk-taking, and humor and has no predetermined goal or destiny [25, 50, 58]. Play is the foremost occupation of a child, but it is important to pursue at any stage of life. Play invites spirit into our lives by conjuring joy, wonder, awe, and inner peace [66]. All forms of play are valuable, and there is no correct or functional way to play [67]. When play is hindered, it prevents us from tapping into the vital force that suspends reality and connects us to human and nonhuman kin. In a suspended state of reality, we connect with our true selves and with our spirit. Allowing and inviting others to play or engage in spiritual practices is a loving act that encourages the integration of knowledge, growth, and development. Through the process of play, people learn what ignites their inner fire, discover their talents, and use this knowledge to share their gifts with their communities to serve the common good.

By facilitating play activities or by encouraging clients to engage in cultural or spiritual practices, therapists can work with clients to help move their trauma energy through their bodies [68]. This can be therapeutic because when trauma energy is lodged within the body, it can cause a person to spiritually disconnect from their body [30]. Trauma can hinder people's engagement in activities that help them reconnect spiritually. Play can help a person find spiritual inspiration and connect with others. Play should never be taken away as punishment and is not a reward for good behavior, it is a fundamental human right [69].

*(2) Pillar 2: Compassion (Social Participation, Adolescence, Emotional, Blood, and Water)*. Women did not have access to clean drinking water. Women organized their communities (*barrios*) and advocated for greater access to resources. They formed mutual aid societies. They prioritized the well-being of others over riches and luxuries. They sought out their citizenship and voted. Adolescents' social participation was often restricted. Women experienced social barriers such as discrimination. They were not treated with compassion by their bosses.

This pillar describes social participation as activities that promote connection with others [25, 50]. Social participation and maintaining healthy relationships across the lifespan are correlated with greater longevity of life [70]. During adolescence, social groups and friends become increasingly important, and therapists can facilitate emotional maturity by instilling compassion, learning to set healthy boundaries, resolving conflicts, and cooperating with others [71]. Compassion is generally understood as an awareness of another's suffering, coupled with the desire and willingness to alleviate another's suffering [72]. Compassion facilitates stability in relationships and connection with others. Cultivating compassion improves relationships, emotional regulation, and mental health. Techniques such as deep breathing and awareness of facial expressions/tone of voice can enhance prosocial behaviors that instill compassion and help us feel safe and cared for.

Water, like compassion, establishes societal norms because societies are built around water, and all beings are interconnected through our relationship with water [73]. Running water, like our emotions, has qualities such as patience, resistance, and tenacity. Running water cleanses and can purify. Water can be calm or turbulent. It can become stagnant, flow, or change quickly. Water can spill over or be contained (in a vessel or dam). Temperance is necessary for humans to maintain equilibrium with water and our emotions.

In the sacred pictorial manuscripts of the Nahua people (codices), water is represented as something precious, a jade stone [74]. Without water, there is no life. Water is the Earth's lifeblood and must be regarded as a relative and sacred gift rather than a property or resource to be exploited. To this extent, we are compelled to care for water, share it, protect it, keep it clean, and honor it [75]. Water can be dangerous; it can drown indiscriminately and should be treated with respect.

The heart is the organ that pumps our blood. The heart is the center of our vitality and intuition, our inner light that sparkles like the sun reflected on water's surface. Water reflects light, shines, and acts as a mirror. Blood pumps from the heart and flows through our veins and arteries, like streams and rivers on the Earth. Our blood is mostly made of water. Water on Earth is never added nor disappears, it is simply transferred from the Earth to our blood. In this way, we share our blood (water) with our ancestors and carry their memories and emotions with us [76].

Therapists can recognize that historical trauma has health implications. We can learn about people's histories and approach our clients using culturally safe, trauma-informed practices [77]. Restall and Egan [78] propose a collaborative, relationship-focused practice that involves sharing power with our clients. This requires therapists to engage clients through a reflexive process that involves a commitment to lifelong learning and a critical self-reflection of how our own positioning impacts the health and healing of our clients.

*(3) Pillar 3: Sustainability (Work, Adulthood, Physical, Body, and Land)*. Women's lives were dominated by work, but they struggled to make ends meet because their work was exploitative and not sustainable. Women and children had to bear the burden of absent fathers/husbands and worked harder to compensate for their absence. Physical labor was required in the mines, fields, and homes. Play, social participation, and education were restricted because of an overemphasis on work.

Work refers to the exertion or labor involved with producing, delivering, or managing services or objects. Work can be paid or unpaid and can include exercise, manual labor, domestic work, or cognitive work [25, 50]. Adults predominately maintain this pillar, but work is important at all stages of life. Work is sustainable when it is nonexploitative and provides fair pay, opportunities, and access to built environments (schools, hospitals, food, and parks).

Many Indigenous philosophies convey the understanding that people emerged from the land and that our bodies are made of the land [63]. Humans cannot be masters of the land or ever be separated because we are the land and dependent upon the land for life. The land gives us medicine, food, and water; therefore, our well-being is dependent on the well-being of the land [65]. Land is the giver and receiver of life [5]. Humans are stewards of the land, and all living and nonliving entities require our care and attention. Understanding that the Earth is alive and has a heart necessitates humans to live sustainably with the land and all its creatures. This requires respect and reciprocity as the fundamental values needed to live sustainably with the land and each other.

Occupational therapy is a sustainable form of healthcare because supporting people in occupational participation is both the goal and the treatment [79]. Therapists can work to improve health and well-being by improving accessibility to resources and opportunities that support occupational participation. It is imperative that therapists renounce medical hegemony and predatory capitalist ventures like the Autism Industrial Complex (AIC) that exploit people's differences by treating their bodies as commodities and prioritizing profits over well-being [80]. Therapeutic interventions that reject “otherness” by claiming to “fix” people and imposing a normative culture should be repudiated [79]. Practitioners can be informed by diverse cultural perspectives that challenge the paradigm from one of taking to one of giving. This change in perspective can have a rippling impact on reducing our carbon footprints and protecting people and the land from exploitation and environmental devastation [81]. Environmental devastation is a threat to culture and biodiversity [82].

The TMOT recognizes that physical and cognitive capacities are always changing and in flux. Abilities can be understood on a spectrum and not dualistically, simply as abled or disabled [83]. Disability is not a deficit because all kin work together to maintain equilibrium. There is no understanding of individuals existing separate from their environment, only an understanding that additional support may be needed in some areas to maintain equilibrium and harmony.

*(4) Pillar 4: Language (Education, Elders, Cognitive, Breath, and Wind)*. Women desired an education and had the cognitive abilities needed to pursue a higher education, but discrimination, geographical distances, lack of family support, and an overemphasis on productive occupations were barriers. Women valued preserving their Spanish language and regretted not learning English. Children were punished and shamed for speaking Spanish in schools. Women reflected on their lives as elders and shared their stories so the younger generations could learn from them.

The occupation associated with this pillar is education. Education includes any process in which people learn, teach, or acquire knowledge or skills. The elders of our communities help us maintain our cultural identity through sharing knowledge and transferring language. Their teachings are precious because they ensure the well-being of future generations [84]. The wind is reflected through our breath and the language we use to tell our stories and share our memories. Oxygen is in the wind we breathe [85]. Breath is shared by all living things, and all living things have a soul [63]. Seeds are carried in the wind, allowing them to propagate and germinate. Knowledge and wisdom are transmitted through breath (language), take root, and grow in our minds and hearts.

Language can be verbal or nonverbal and can include symbols and gestures. Depriving people of their language threatens linguistic diversity and can be weaponized to disempower and exploit vulnerable people. Practitioners must be critical of monolingual ideologies that encourage hegemony through imposing and restricting language [86]. Language justice necessitates welcoming multilingual spaces so that many voices can be heard and considered equally. Therapists can work towards supporting clients in accessing resources in their preferred language to increase their agency within education and healthcare settings. Responding to each person's unique language needs welcomes diverse identities. Honoring all forms of language and communication is vital to promoting people's agency and validating that what they do and say matters and has an impact. We can ensure that all participants have the necessary resources to engage in the learning process and acquire new skills [86].

When societies work together to keep the Four Pillars of Culture in equilibrium, by contributing to the common good through work, social participation, play, and education, health can be achieved for all interrelated systems. Individual and environmental health is understood as a reciprocal and dynamic relationship between a microcosm (individual) and macrocosm (environmental). The essence of human agency, within this context, is when humans understand that their behaviors in the microcosm impact their larger macrocosm and vis-à-vis. Health within this model is not viewed as the absence of illness but as the maintenance of equilibrium and balance with the Earth and all our kin through practicing the Four Pillars of Culture (self-determination, compassion, sustainability, and language) and engaging in transformative occupations that serve the common good.

### 3.4. Limitations

The women's stories do not represent the experiences of all Mexican Americans, and the findings may not be generalized. The women have passed away, and their stories were published years after their deaths. There was not an opportunity to ask more questions or gain clarity and insight into their experiences. The women were taken from a convenience sample who may have chosen to omit details about themselves. There is a lack of representation from the LGTBQ+ community, or women did not discuss their gender expression or sexual orientation. The stories were translated from Spanish to English, and some nuances of their stories were consequently lost in the translation.

This work reflects myself, my community, my ancestors, and others within my profession. It is an expression of my cultural identity and reflects my perspective as a pediatric occupational therapist, Xican@, daughter, mother, and wife. My insights can expand and change, and I am open to other interpretations or critiques. This work is meant to incite dialogue and foster a paradigm shift. Cross-cultural research is needed to expand upon current theories within occupational therapy and derive a more robust understanding of how practitioners can support clients in engaging in health-sustaining occupations that support their happiness, well-being, and quality of life.

## 4. Final Considerations

Occupational therapists can raise their occupational consciousness by recognizing how environmental barriers such as pollution, exploitation, and discrimination impact occupational participation. Therapists have a responsibility to understand individuals within the context of their environment and support healing within families, communities, and populations [78]. Cultural humility requires practitioners to reflect on how our implicit biases and behaviors maintain oppressive systems. Advocating for equal access to health-promoting occupations such as social participation, play, education, and sustainable work opportunities, while renouncing privileged assumptions that productivity and leisure contribute positively to health, is an act of resistance that occupational therapists can exercise in their everyday practice. Helping our clients exercise their agency within their own small acts of resistance against hegemony is also indicated.

The stories highlight the values and behaviors that sustain the women's communities and challenge stereotypes about Mexican women being martyrs, docile, or passive. Their inspiring *si se puede* (yes, we can) attitude, first coined by Dolores Huerta, cofounder of the United Farm Workers Union, is pervasive. It can be seen in their views of using welfare as a last resort, finding creative ways to make money, community organizing, obtaining citizenship, and helping one another in times of need. Within the occupational therapy profession, strength-based approaches that support people's cultural values should be highlighted rather than engaging in deficit discourse [46, 87].

The reflexive process of remembering the women's stories and integrating their wisdom and insights into our profession's historical memory can recast a diverse profession that considers the voices and experiences of all people. The past is not recorded and static but is pliable and can be rewritten in the present. Cross-cultural theories like the TMOT are needed to conceptualize a holistic, sustainable, and inclusive profession that honors self-determination, compassion, sustainability, and language. As a Xican@, theorizing validates knowledge rooted within my ancestral memory. Occupying this theorizing space allows for reconciliation and healing and gives validity to the everyday lives of the people who built and created our cultural landscapes in the agricultural fields, mining towns, and *barrios* [88, 89].

## Data Availability

Data is available upon request through the authors of *World of Our Mothers: Mexican Revolution–Era Immigrants and Their Stories* [1]. Contact Miguel Montiel and Yvonne de la Torre Montiel to request the data including cassette recordings and oral and written transcriptions or the recorded interviews. The authors can be contacted at the following email address: miguelmontiel@asu.edu.
